# ROS Consumers or Producers? Interpreting Transcriptomic Data by AlphaFold Modeling Provides Insights into Class III Peroxidase Functions in Response to Biotic and Abiotic Stresses

**DOI:** 10.3390/ijms24098297

**Published:** 2023-05-05

**Authors:** James New, Daniel Barsky, Claudia Uhde-Stone

**Affiliations:** 1 Department of Biological Sciences, California State University, East Bay, Hayward, CA 94542, USA; jnew@horizon.csueastbay.edu; 2 Department of Physics, California State University, East Bay, Hayward, CA 94542, USA; daniel.barsky@csueastbay.edu

**Keywords:** AlphaFold, Arabidopsis, class III peroxidases, plant stress response, phosphate deficiency, ROS signaling

## Abstract

Participating in both biotic and abiotic stress responses, plant-specific class III peroxidases (PERs) show promise as candidates for crop improvement. The multigenic PER family is known to take part in diverse functions, such as lignin formation and defense against pathogens. Traditionally linked to hydrogen peroxide (H_2_O_2_) consumption, PERs can also produce reactive oxygen species (ROS), essential in tissue development, pathogen defense and stress signaling. The amino acid sequences of both orthologues and paralogues of PERs are highly conserved, but discovering correlations between sequence differences and their functional diversity has proven difficult. By combining meta-analysis of transcriptomic data and sequence alignments, we discovered a correlation between three key amino acid positions and gene expression in response to biotic and abiotic stresses. Phylogenetic analysis revealed evolutionary pressure on these amino acids toward stress responsiveness. Using AlphaFold modeling, we found unique interdomain and protein–heme interactions involving those key amino acids in stress-induced PERs. Plausibly, these structural interactions may act as “gate keepers” by preventing larger substrates from accessing the heme and thereby shifting PER function from consumption to the production of ROS.

## 1. Introduction

Plants are increasingly exposed to both biotic and abiotic stresses [1], a problem exacerbated by climate change. To prevent global yield losses, a better understanding of the mechanisms by which plants cope with multiple stresses is needed. Class III peroxidases (EC 1.11.1.7, PERs) are plant-specific heme oxidoreductases at the crossroads of biotic and abiotic stress responses and have been put forward as promising candidates for crop improvement [2]. In plants, *PERs* have evolved into large gene families, with over 150 members in the bryophyte Marchantia, 138 in rice, and 73 in Arabidopsis [3].

Such a large number of genes, combined with wide variations in their spatiotemporal expression, suggests functional specializations [4,5,6]. Indeed, many substrates of PERs have been identified, including lignin precursors, auxin, and secondary metabolites [4,5,6]. Prior research has shown that PERs function in cell wall metabolism, cell elongation, wound healing, auxin metabolism, seed germination, defense against pathogens, and signaling in response to biotic and abiotic stresses [5,6]. However, linking these functions to specific PERs has proven difficult, in part because, in vitro, these peroxidases show low substrate specificity.

Most PERs are secreted into the apoplast, where they are thought to modulate levels of apoplastic ROS (reactive oxygen species) both positively and negatively [4,6]. The traditional view of peroxidases holds that they oxidize a substrate, such as a lignin precursor while reducing H_2_O_2_ to H_2_O. However, PERs can also produce ROS: hydroxyl radicals (OH^•^) in the hydroxylic cycle and superoxide anions (O_2_^•−^) in the oxidative cycle. The superoxide anion is immediately converted to H_2_O_2_ and O_2_ either spontaneously or through superoxide dismutase [7]. This generation of free radicals can have various and sometimes contrasting functions, such as cell wall stiffening by promoting covalent bonds or cell wall loosening due to the breakdown of polysaccharides [6,8,9].

Interestingly, PERs function in both stress reception and responses, and specific PER expressions are induced by various biotic and abiotic stresses, such as pathogens, metals, ozone, temperature, anoxia, and nutrient deficiencies [5,10]. PERs are also known to play a central role in plant defense both by cell wall cross-linking and by producing large amounts of toxic ROS in an oxidative burst—part of the hypersensitive response in plants [5,11,12]. In addition, ROS are known to act in signal transduction pathways in defense against pathogens [2,5,12]. Evidence is accumulating that ROS-producing PERs are also involved in the signaling of abiotic stress, such as metal toxicity and phosphate (P_i_) deficiency [2,4,5,13]. We hypothesize that PERs that are upregulated in response to biotic and short-term abiotic stresses are likely producers of ROS, either in defense against pathogens or as part of a signal transduction pathway.

Even in evolutionarily distant plant families, PER protein sequences of both orthologues and paralogues are highly conserved, yet the discovery of correlations between sequence differences and their functional diversity has proved difficult [3,14]. Models of enzymatic mechanisms developed over the last century were greatly advanced with the advent of X-ray crystallographic structures in the 1990s. The first PER crystal structure solved was in peanut [15], quickly followed by a horseradish PER [16]. Of the 73 PER paralogues in Arabidopsis, only the structures of PER53 (alternative name ATPA2) [17] and PER59 (alternative name peroxidase N) [18] have been crystallographically determined. As expected, the crystal structures are all very similar and confirmed earlier models [19] that show that PERs are composed of two largely α-helical domains, one “distal” and one “proximal”, each of which binds a calcium ion, as well as a third, smaller “β-domain”. The structure is stabilized by a network of polar and non-polar interactions orienting the heme, four to five conserved intra-domain disulfide bridges, and the two Ca^2+^ binding sites [20,21,22,23,24].

Despite the available structures, previous modeling approaches did not reveal clearly distinguishable structural features among PERs that linked them causally to the production or consumption of ROS [5,25]. With the emergence of AlphaFold, a recent breakthrough in protein structure modeling [26,27,28], we saw a much-improved opportunity to analyze structural differences that might be responsible for some of the functional diversity among PERs.

In this paper, we combine transcriptomic meta-analysis, bioinformatics—including multiple sequence alignment, motif search, and phylogenetic analysis—and AlphaFold modeling to identify key differences within the Arabidopsis PER family that potentially have structural/functional consequences related to ROS production or consumption.

## 2. Results

### 2.1. Transcriptomic Meta-Analysis Reveals Similarity of Gene Expression Patterns between Biotic and Short-Term Abiotic Stress Responses

Because of the many experiments performed on Arabidopsis and because all 73 class III peroxidase (*PER*) paralogues in this model plant have been identified [21], we confined our analyses, for the most part, to an examination of the gene expression patterns of *PERs* in Arabidopsis.

**Figure 1:**
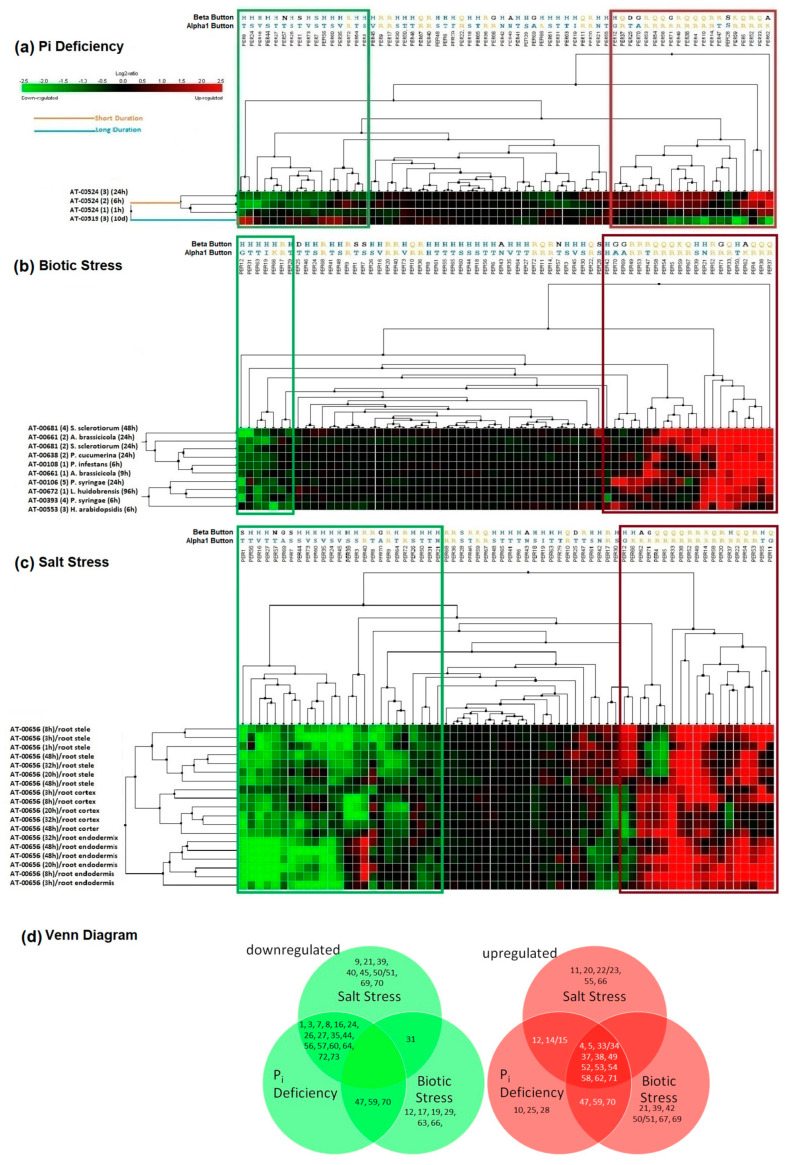
(**a**–**c**) Hierarchically clustered heatmaps generated with Genevestigator reveal differential gene expression of Arabidopsis *PERs*. Upregulated genes are indicated by red, and downregulated by green boxes. Pairs of letters above each gene indicate amino acids in two positions as explained in the text. (**a**) Root response to P_i_ deficiency, showing an almost opposite pattern of differential gene expression in short-term (1 h, 6 h, and 24 h; top 3 rows), compared to long-term P_i_ deficiency (bottom row). (**b**) Root responses to various pathogens (biotic stresses). Biotic stresses include infection with *Sclerotinia sclerotiorum*, *Alternaria brassicicola*, *Plectosphaerella cucumerina*, *Phytophthora infestans*, *Pseudomonas syringae*, *Liriomyza huidobrensis*, and *Hyaloperonospora arabidopsidis*. (**c**) Root responses to salt stress. (**d**) Venn diagrams visualizing shared upregulated and downregulated genes in response to biotic stresses, short-term P_i_ deficiency, and salt stress.

To compare expression patterns of the 73 *PER* paralogues in Arabidopsis, we used Genevestigator, a curated collection of normalized and systematically annotated transcriptomics data that enables a comparison of the expressions from thousands of experiments, including a vast selection of studies involving biotic and abiotic stresses [29]. While Genevestigator contains both Affymetrix and RNA-seq data, we found Affymetrix data better suited for cross-experimental comparisons, likely because of standardized protocols and the use of identical gene chips, facilitating data normalization [30]. When we compared gene expression data from Affymetrix chip-based experiments of all 73 *PER* paralogues, we noticed a striking similarity in the pattern of upregulation in responses to short-term phosphate (P_i_) deficiency, salt, and abiotic stress (Figure 1a–c). In contrast, the expression pattern switched to an almost reciprocal pattern in the P_i_ deficiency long-term response (Figure 1a). Beyond these stresses, we also noticed similar differential *PER* expression during callus formation (Supplementary Figure S1).

Our Genevestigator-powered meta-analysis, summarized in Venn diagrams (Figure 1d), revealed 13 *PERs* upregulated in response to all three stresses (short-term P_i_ deficiency, salt, and biotic stress). In contrast, 16 *PERs* were downregulated both in short-term P_i_ deficiency and salt stress. No *PERs* were downregulated under all three stresses.

### 2.2. Bioinformatic Analysis Reveals Three Key Amino Acid Positions That Determine Membership in Either of Two Groups of PERs: Those That Consume and Those That Produce H_2_O_2_/ROS

**Figure 2:**
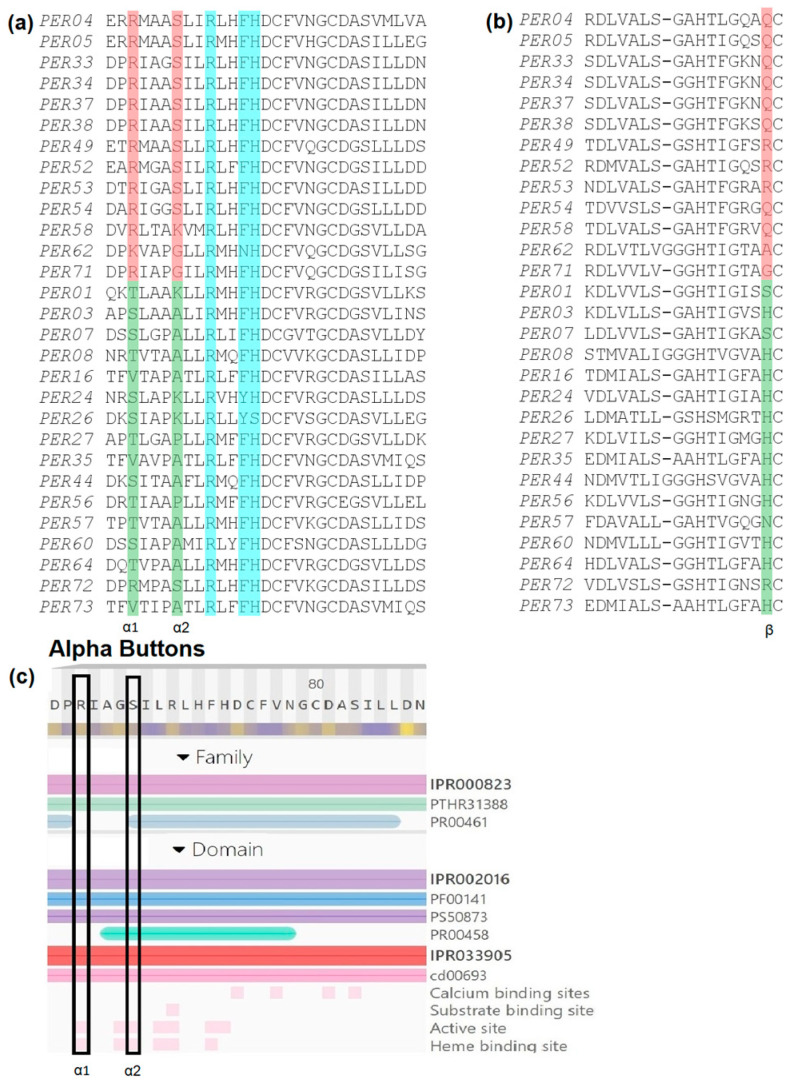
(**a**) MSA of common stress-upregulated (top 13 sequences) and downregulated (bottom 16 sequences) PER amino acid sequences reveals a difference between both groups at the two *Alpha Buttons*. The amino acid position is shaded in red for upregulated and green for downregulated *PER* genes. In cyan, three highly conserved active site residues are also indicated. (**b**) The same MSA revealed another conserved difference at the end of the first of two beta strands, at a position we termed the *Beta Button*, shaded as for the *Alpha Buttons*. (**c**) Support for the significance of the two *Alpha Buttons*: a position-specific scoring matrix, part of an InterProScan search, revealed both *Alpha Button* positions (marked by a black rectangle) as likely having a role in the active site and in heme binding.

To look for key differences between the peroxidases that are upregulated versus those downregulated in response to biotic and short-term abiotic stress, we created a multiple sequence alignment (MSA) with amino acid sequences of the 13 stress-upregulated and the 16 stress-downregulated PERs (Figure 2a,b).

Examining the MSA, we found several amino acid sites that were conserved within each group (up- or downregulated) but differed between both groups. Specifically, and significantly for our analysis, we identified three key positions that we dubbed *Alpha1*, *Alpha2*, and *Beta Buttons*. At the *Alpha1 Button* (Figure 2a), most stress-induced peroxidases contain an arginine (R), while most downregulated peroxidases contain either a threonine (T), serine (S), or valine (V). At the *Alpha2 Button* (Figure 2a), most stress-induced PERs have an S, while most downregulated PERs have an alanine (A). Finally, in the *Beta Button* position, the upregulated PERs usually have an R or glutamine (Q), while the downregulated PERs typically have a histidine (H) in that position (Figure 2b). Expanding our analysis to all 73 PERs, we found that 28 peroxidases have either R, Q, or lysine (K) in the *Beta Button* position, while 33 peroxidases contain a H in this position.

In addition to the MSA, we used InterProScan to identify functionally significant sites in these PERs. InterPro combines protein signatures from 13 databases, including the Conserved Domain Database (CDD) at NCBI, which uses a position-specific scoring matrix (PSSM). In this case, the CDD results provided additional evidence that both the *Alpha Buttons* are significant and may play a role in both the active site and heme binding (Figure 2c).

In summary, we found that stress-induced PERs tend to contain R or K at the *Alpha1 Button*, S at the *Alpha2 Button*, and R or Q at the *Beta Button*. Conversely, downregulated PERs tend to contain T, S, or V at the *Alpha1 Button*, A or K at the *Alpha2 Button*, and H at the *Beta Button*. The *Alpha1* and the *Beta Buttons* for each *PER* are listed in Figure 1 across the top of each profile (for simplicity, only these two out of the three “buttons” are shown), displaying, along with any exceptions, the strong correlation of these buttons with gene expression patterns. In the next section (Section 2.3), we will see that there also appears to be a strong phylogenetic correlation with PER stress-response regulation and, moreover, with the three buttons identified above. Lastly, in Section 2.4, we will see that analysis of the 3D structure allowed us finally to infer the functional significance of these buttons.

### 2.3. Phylogeny Reveals Correlation between Gene Expression and Evolutionary Relationship

**Figure 3:**
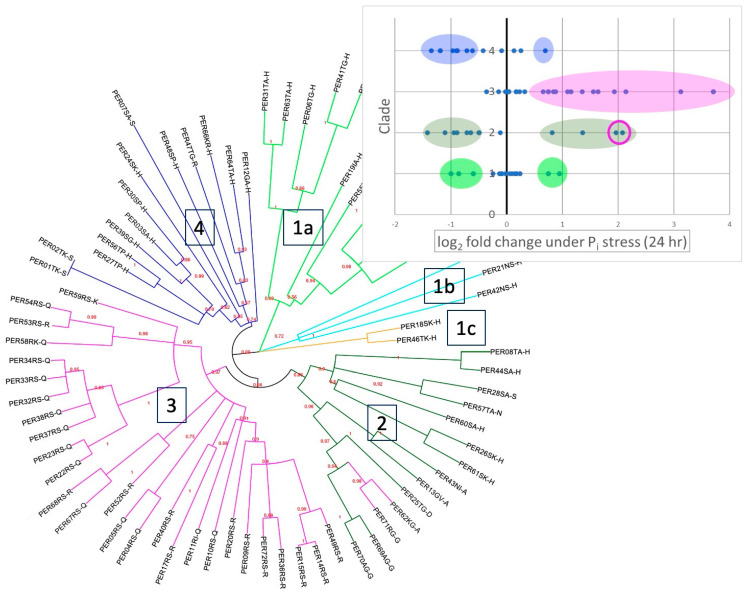
Maximum likelihood tree based on the 73 PER coding RNA sequences. The four main clades are distinguished by color and labeled 1−4. Approximate likelihood-ratio values are shown in red near the corresponding nodes. The particular amino acid at the *Alpha1* and *Alpha2 Buttons* and the *Beta Button* are noted at the end of each protein name, using the single letter code. Note that Supplementary Figure S2 shows all three buttons in a rectangular phylogenetic tree for better legibility. The insert shows gene expression as log2 fold change under short-term (24 h) P_i_ deficiency, with ±0.5 log2 fold differentially expressed genes, highlighted by color shading. Circled in magenta are PER62 and PER71, which we postulate have convergently evolved to function like members of clade 3.

As mentioned, the level of conservation among the multigene family of Arabidopsis class III peroxidases (PER) is quite high; by pairwise alignment, we found roughly 40% to 70% sequence identity in PER sequences—even higher among recently duplicated genes. (For example, the amino acid sequences of the mature proteins PER33 and PER34 are 94% identical and in fact cannot be distinguished in microarray analysis.) In order to look for possible correlations between observed expression differences and phylogeny, we generated a maximum likelihood tree of all 73 PERs. We based this tree on an MSA of RNA-coding sequences rather than amino acid sequences because the high conservation of PER proteins made an amino acid-based MSA less robust. To assess the reliability of nodes, we used the aLRT (approximate likelihood-ratio test) [31] (Figure 3; rectangular version available in the Supplementary Material as Figure S2), which provided higher confidence scores for the larger clades than traditional bootstrapping.

The resulting tree (Figure 3) contains four large clades (clades 1, 2, 3, and 4). Interestingly, we found a strong phylogenetic correlation with gene expression in response to short-term P_i_ deficiency in clades 3 and 4: most members of clade 3 (Figure 3, magenta) are upregulated in short-term P_i_ deficiency, while most members of clade 4 are downregulated (Figure 3, blue). Clades 1 and 2 include both upregulated and downregulated genes.

While the pattern of *Alpha* and *Beta Buttons* described in Section 2.2 generally also correlate with the phylogenetic clades, there are two interesting exceptions: PER71 and PER62 belong to clade 2 yet have residues in the *Alpha1 Button* that are typical for clade 3. Moreover, in contrast to other members of clade 2, the expression pattern of PER71 and PER62 resembles that of clade 3, with both members being highly upregulated in response to short-term P_i_ deficiency and biotic stresses (circled in the inset graph of Figure 3).

### 2.4. Crystal Structures and AlphaFold Modeling Reveals the Alpha and Beta Buttons as Possible Gate Keepers That Modulate Access to the Heme

To identify possible structural implications of the *Alpha1*, *Alpha2*, and *Beta Buttons*, we sought to put the newly available 3D structural models of PERs created through AlphaFold together with the crystal structures of PER53 [17] and PER59 [18]. According to Alphafold, the confidence levels of the models are overall very high (shown in Supplementary Figure S3), except for the N-terminal signal peptides, which are not part of the mature protein.

To make detailed comparisons, we used the PER53 crystal structure as a reference and performed structural pairwise alignments of PER models to PER53 (PDB code: 1pa2) using the LGA program [32,33]. To put these structural alignments in perspective, we also aligned the AlphaFold model of PER53 as well as the crystal structure of PER59. All the resulting alignments—each pairwise to PER53 (1pa2)—were achieved with nearly all residues included and at low overall RMSD (root-mean-square deviation, listed in Table 1). A true prediction (not simply a copy of experimental data), the AlphaFold model of PER53 nevertheless fit the crystal structure of PER53 very tightly with an RMSD of 0.25 Å, meaning that the average backbone deviation was only a quarter the diameter of a hydrogen atom. More astonishing, however, is that the PER33 model fits the crystal structure of PER53 also very tightly, slightly more tightly even than the crystal structure of PER59!

**Figure 4:**
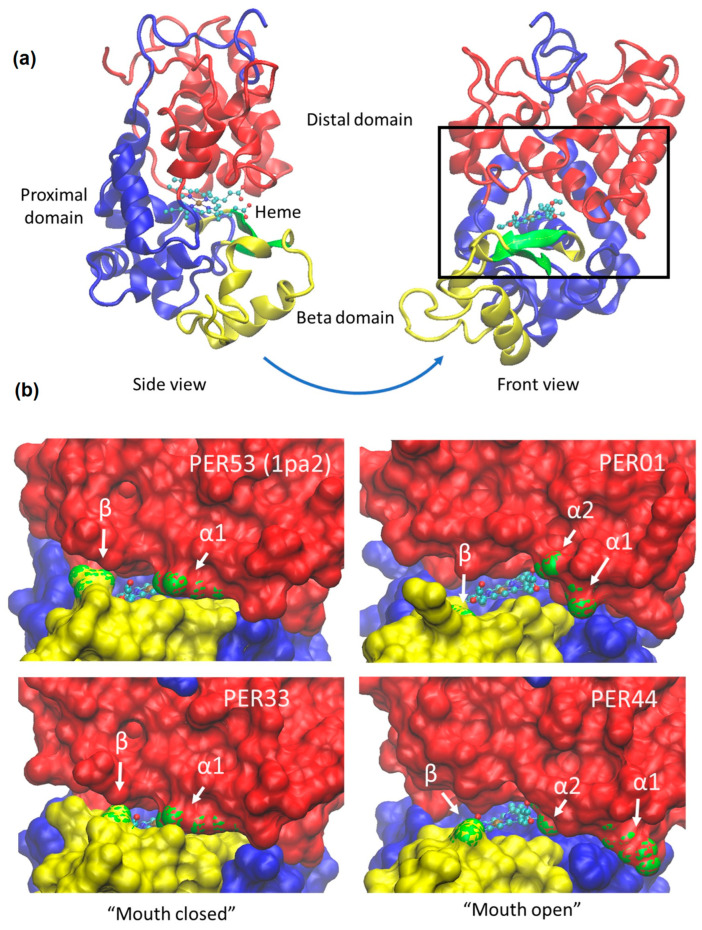
(**a**) Cartoon view of PER53 based on the crystal structure [17], with bound heme (ball and stick model) located between the distal domain (red), the proximal domain (blue), and the beta domain (yellow and green). The heme propionate groups, each ending with a pair of oxygen atoms (red balls), emerge from the γ-edge of the heme (to the right in the side view, toward the viewer in the front view). (**b**) “Front” views into the “mouth” of PER AlphaFold models and PER53 crystal structure with coloring and orientation as in Figure 4a (black rectangle), except here the green highlights are *Alpha1* (α1), *Alpha2* (*α2*) and *Beta* (*β*) *Button* residues (indicated by arrows). In the left column (PER53 and PER33,) *Alpha2* is occluded from view, and the mouth is less open. In the right column (PER01 and PER44), the mouth is more open, as evidenced by more of the bound heme being visible. We hypothesize that the less open mouth restricts access to the heme for larger substrates while allowing access for small molecules such as H_2_O_2_, H_2_O, •OH, O_2_•^−^, etc.

The crystal structures also contain a bound heme group (“heme b” or protoporphyrin IX), which is required for the functioning of all peroxidases (Figure 4a). Given the very high structural similarity of the PER models to the PER53 crystal structure, we included the heme in our PER models by simply copying the atom coordinates from the PER53 structure after the alignment. This way of modeling the bound heme worked exceedingly well as judged by only a single, near clash in PER44. (The gamma carbon of an isoleucine side chain (I183) was positioned within 1.4 Angstroms of a terminal carbon atom on the α edge of the heme. This near clash is of no consequence as it could have been relieved by a tiny adjustment of the isoleucine side chain or by a fleeting application of molecular dynamics. No other clashes were reported in any of the models examined here.) Protruding from the fourth (γ) edge of the heme are two negatively charged propionate groups. Significantly, studies have suggested that compounds oxidized by PER (such as lignin precursors) require access to a heme edge or to the propionate groups [34]. 

As mentioned, the PER structure entails a “distal” and a “proximal” domain, as well as a third, smaller “β-domain” (Figure 4a). A useful analogy is that the overall protein structure resembles a mouth, where the heme plays the role of the tongue and where the β-domain functions as a lower lip that can meet the propionate tips of the tongue.

Using the crystal structures and the AlphaFold models, we found that, although the *Alpha Buttons* are both separated by about 150 amino acids from the *Beta Button* in their primary structures, in their tertiary structures, all three buttons are in close proximity and at the opening of the “mouth” of the active site. Structural analysis allows us to propose a similar role for all of the three buttons, resulting in either a “closed mouth” or an “open mouth” conformation. In particular, by analyzing members of both groups, we found that peroxidases with either an R or Q in the *Beta Button* tend to form a hydrogen bond with the near heme propionate group (on the left in Figure 4a, front view, shown in the Supplementary Material), leading to a “closed mouth” conformation (Figure 4b, left panels), while the peroxidases with an H in the *Beta Button* do not form such bonds, because the amino acid is too short to reach the heme propionate tips, leading to an “open mouth” conformation (Figure 4b, right panels). A similar analysis of the *Alpha Buttons* leads to a similar conclusion. In summary, the bigger polar side chains R, Q, and K of the *Alpha1* and *Beta Buttons* and the smaller polar S at the *Alpha 2 Button* are able to form hydrogen bonds that “button up” the entrance to the substrate binding pocket, and thus likely restrict access of larger substrates to the heme and its propionate groups. Stereo views of the hydrogen bonding details described here are presented in the Supplementary Materials (Figure S4). These results are summarized in Table 2.

## 3. Discussion

What distinguishes the many PER paralogues from each other, and what causes some PERs to consume and others to produce ROS? To address these questions, we started here with an exploration of the expression patterns of Arabidopsis class III peroxidase genes (*PERs*) in roots during cellular responses to biotic and short-term abiotic stress, specifically P_i_ deficiency and salt stress. Several of the genes that we have identified here as upregulated have been shown in prior research to be induced in response to pathogen attack and P_i_ deficiency, namely *PER33/34* (too similar to be distinguished in the microarray analysis), *PER62*, and *PER71* (reviewed in [5]).

We have been particularly interested in the contrasting functions of H_2_O_2_-consuming and ROS-producing peroxidases. These differences may be caused by variations in reaction conditions, particularly the availability of suitable substrates, or by structural differences in the enzymes. It seems likely that *PER* genes that are induced by stress, especially by biotic stresses, tend to be producers of ROS, as ROS production is a well-documented response to pathogen and insect attacks. Such correlation is further supported by reverse genetics revealing PER33/34 (induced by biotic and abiotic stresses) as ROS producers [35]. Comparing up- versus downregulated PERs by MSA, we found a strong correlation between gene expression and the three key amino acid differences, which we have termed the *Alpha* and *Beta Buttons*.

Using AlphaFold modeling, we discovered evidence of structural variations that correlate with PER expression patterns in response to biotic and abiotic stresses and can be understood through the structural actions of the buttons. The AlphaFold models specifically revealed that the group of stress-induced PERs (likely ROS producers) tend to have hydrogen bonds involving the *Alpha* and *Beta Buttons* that “button up” the entry to the substrate access channel, which could prevent access by larger substrates to the heme, preventing their oxidation. This conclusion is supported by the finding that PER33, PER34, and separately PER53—all “closed mouth” PERs—have been shown to produce H_2_O_2_ bursts in response to biotic stress [36,37]. On the other hand, PERs that are not stress-induced (likely H_2_O_2_ consumers) tend not to form these hydrogen bonds, thereby allowing larger substrates access to the heme. These findings are in agreement with early thoughts on the subject by Schuller et al. (1996), who suggested that the differential functioning of class III peroxidases may be caused by changes in the access to the active binding site through the substrate access channel [15].

The crystal structure of the putative PER53 orthologue in switchgrass revealed several protein–heme hydrogen bonds involving six amino acids [24]; three of those correspond to the *Alpha* and *Beta Buttons* identified here. Although the other three may turn out to also play a role in PER function in terms of ROS production or consumption, they did not stand out in our analysis as being strongly correlated with expression or clade. Ironically, in addition to PER53, the only other crystal structure of Arabidopsis PERs is PER59 (N) which is also a “closed mouth” PER. It is worth emphasizing that this has meant that, without the AlphaFold models, we would not have been able to observe any “open mouth” isoforms. Furthermore, the apparent accuracy of the AlphaFold models combined with the high structural conservation of the PER family has allowed detailed analysis of the role of the key identified residues. In addition to the importance of the three buttons identified and the modeling detail at the level of hydrogen bonds, we want to also point out that the open vs. closed mouth conformation that is shown in Figure 4b appears to extend somewhat beyond the side chains. In Table 1, we tabulated the tightness of the structural alignments, and while all the RMSD values are reassuringly low, we also notice that on the one hand of the three AlphaFold models, PER33 is the closest to PER53, which makes sense given that PER33 and PER53 are, by our analysis, both “closed mouth” isoforms. On the other hand, also as one would expect, the two least tightly fitting models are both “open mouth” isoforms: PER01 and PER44.

Unfortunately, there is a dearth of data about which PERs produce or consume ROS. As mentioned above, PER33, PER34, and PER52 have been shown to produce ROS [5,36,37] and are in support of our model. PER57, however, which showed downregulation in both early P_i_ and salt stress in our meta-analysis, has been reported to produce ROS [38], contradicting the strong correlation with regulation. PER71, an interesting case of possible convergent evolution, has been described as a likely producer of H_2_O_2_/ROS, as revealed by reverse genetics [39], but it has also been described as an enzyme involved in lignin production, a reaction that consumes H_2_O_2_ [6]. This possibly dual nature may indicate that some PER may at different times act in more than one catalytic cycle, depending possibly on environmental factors, particularly the availability of a suitable substrate.

Despite these possible exceptions, the key residues that we have identified in the Arabidopsis PER family on the basis of expression profiles were shown here to have plausible roles in buttoning up or leaving open the enzyme, i.e., allowing or restricting access of large molecules to the heme. Broadly, it appears that the tendency of a PER to be closed mouth versus open mouth is coded into the sequence of the individual PERs and that this, in turn, controls H_2_O_2_ consumption versus ROS production. Forthcoming biochemical experimentation, as well as crystal structures of consumer PERs, should further illuminate this.

## 4. Materials and Methods

### 4.1. Meta-Analysis of Gene Expression

Beginning on 11 August 2021, we employed Genevestigator (https://genevestigator.com) (v9.6.1), a curated database of transcriptomic data for meta-analysis [29,30] to examine the expression patterns of Arabidopsis class III peroxidases (*PERs*) in response to stress. To this end, all 73 Arabidopsis *PER* locus numbers were obtained from TAIR (https://www.arabidopsis.org) and used to produce a complete gene data set within Genevestigator. We then selected the Affymetrix platform (ATH1) data sets and focused on experiments of plant roots under various biotic and abiotic stresses.

Based on our previous research and literature searches, we were especially interested in experiments that showed differential gene expression in response to abiotic stresses in roots. Comparing the many available experiments in response to abiotic stress, we noticed that short-term P_i_ deficiency and salt stress studies showed especially high differential gene expression among *PERs*. Based on this observation, we selected the P_i_ deficiency experiments At-00524 and At-00519, which were specific to roots and included early (1 h, 6 h, and 24 h) and late (10 days) time periods of P_i_ deficiency. For salt stress, we selected At-00656, which was also specific to roots and had similar time points (1 h to 48 h) compared to the early P_i_ deficiency experiments. For biotic stress, we selected a sample of eight experiments that had similar time points (6 h to 96 h) to the early P_i_ deficiency and salt stress studies (At-00106, At-00108, At-00393, At-00553, At-00638, At-00661, At-00672, At-00681).

### 4.2. Multiple Sequence Alignment (MSA) and Site-Specific Analysis to Identify Key Amino Acids

We used the MSA editor Jalview (v2.11.2.5) [40] to edit the amino acid sequences of all 73 PERs. We trimmed the signal peptides and the variable tails from all 73 class III AtPERs and removed one large insert in a single sequence (PER48). The edited FASTA sequences are provided as supplementary data in the Supplementary File.

To identify key amino acids correlated to gene expression, we used MUSCLE [41] with default settings for protein alignment, employing a BLOSUM62 matrix to perform an MSA of the amino acid sequences of the 13 PERs upregulated under all three stress conditions (P_i_ deficiency, salt, and biotic stress), and the 16 PERs downregulated under both P_i_ deficiency and salt stress (Figure 1d) [41].

Finally, we sorted all 73 AtPER amino acid sequences so that sequences with an R in the *Alpha1 Button* were at the top of the list, then used MUSCLE (v3.8.31) to produce an MSA of all 73 PERs (Figure S5).

To further identify positions of functional importance, we submitted a representative sample of those 29 PER sequences to InterPro (v87.0) (https://www.ebi.ac.uk/interpro), scanning for protein signatures from 13 databases, including the Conserved Domain Database (CDD) at NCBI, Panther, Pfam, and Prosite [42].

### 4.3. Phylogeny

We used Phylogeny.fr (http://www.phylogeny.fr/index.cgi) to run alignments in the system’s “A la carte” mode [31]. We employed MUSCLE again to generate an MSA and to produce a maximum likelihood phylogenetic tree [43]. In this case, because of the high conservation of PER proteins, we used RNA-coding sequences from the 73 PERs rather than amino acid sequences. We constructed a phylogenetic tree using the PhyML program (v3.1/3.0 aLRT). Reliability for internal branches was assessed using the aLRT test (SH-Like) [44]. We collapsed branches with support values smaller than 50%. The visualization of the phylogenetic tree was produced using TreeDyn (v198.3) [45].

### 4.4. Structural Analysis of Crystal Structures and AlphaFold 3D Protein Models

To identify the structural roles of the *Alpha* and *Beta Button* residues, we downloaded models from the AlphaFold Protein Structure Database (v2.1.1) (https://alphafold.ebi.ac.uk) [28], which has produced models of a vast number of proteins using the AlphaFold2 program [27]. Choosing several representatives from the upregulated and downregulated groups, we examined the structural models near the “mouth”, particularly access to the tips of the “tongue”, which are formed by the propionate groups of the bound heme.

We performed structural alignments and analysis of the models as follows:

(1) We removed the N-terminal signal peptide regions from the downloaded models (typically 20 to 40 residues). N-terminal signal peptides are removed in the formation of the mature (secreted) protein but were nevertheless present in all the models. In all the models inspected, these peptides protruded unnaturally and were visually removed, and these identifications were additionally supported by the signal peptide program SignalP (v6.0) [46].
(2) The models were pairwise structurally aligned to the crystal structure of PER53 (PDB code: 1pa2) [17], using LGA [32,33]. The alignments were obtained through “local-global” structural comparisons without a preassigned residue correspondence (option 4) and with the default distance cutoff of 5 Å. The coordinates of the heme molecule were simply copied over to the model from the 1pa2 coordinate file after the structural alignment of the model.
(3) The aligned models were analyzed using the VMD program [47], which enabled us to check for clashes, identify hydrogen bonds (protein–protein and between protein and heme), as well as to visualize the mouth and heme accessibility (see Figure 4 and Supplementary Figure S4).
(4) In order to make the correct correspondence of the buttons in each PER, we used the following simple motifs based on our MSA (see Supplementary Materials). These motifs are valid for the entire Arabidopsis PER family:
  - *Alpha1* and *Alpha2*: 1xxx2xxRxxfhDC, where 1 and 2 correspond to *Alpha1* and *Alpha2*, respectively, and RxxfhDC is the highly conserved active site. Uppercase letters are 100% conserved, lowercase letters are mostly conserved, and “x” is variable.
  - Beta: HtxGxxBCxxxxxR, where B corresponds to the *Beta Button*.

## Figures and Tables

**Table 1:** The pairwise alignment of the PER models created by AlphaFold, as well as the PER59 crystal structure, to the PER53 crystal structure. In each case, the reported RMSD is a measure of the overall deviation of the two backbones based on the corresponding C_α_ atoms of aligned residues (see Section 4).

Model	RMSD (Å)	Residues Aligned
PER01	1.42	295
PER33	0.85	304
PER44	1.25	289
PER53 (AlphaFold)	0.25	303
PER59 (PDB code: 1qgj)	1.05	297

**Table 2:** A summary of the frequency of amino acids at each of the three buttons, along with the observed correlation with regulation and mouth posture/heme access. Next to the single-letter amino acid codes are the number of PERs that contain that amino acid in that position, culled from our MSA (see Methods and Supplementary Figure S5). Amino acids that appear most often are in bold; those in only one or two PERs are in lowercase.

Stress-Regulation	Mouth Prediction	*Alpha1 Button*	*Alpha2 Button*	*Beta Button*
Upregulated	Closed	**R**(12), k	**S**(10), g, k	**Q**(8), **R**(3), a, g
Downregulated	Open	**S**(6), **T**(6), V(3), r	**A**(10), K(3) *, p, s	**H**(12), s, n, r

* Although lysine (K) is a polar residue, it apparently cannot make a hydrogen bond with the heme because (in contrast to serine) the lysine side chain is too long, given the position of the protein backbone relative to the heme. We, in fact, observe this in our model of PER01 (see Supplementary Figure S4).

## Data Availability

Not applicable.

## Supplementary Materials

The following supporting information can be downloaded at: https://www.mdpi.com/article/10.3390/ijms24098297/s1.
