# Supplementary figures and images for: ROS Consumers or Producers? Interpreting Transcriptomic Data by AlphaFold Modeling Provides Insights into Class III Peroxidase Functions in Response to Biotic and Abiotic Stresses
Source: Int J Mol Sci. 2023 May 5;24(9):8297. doi: 10.3390/ijms24098297 (PMC10179425; doi:10.3390/ijms24098297)

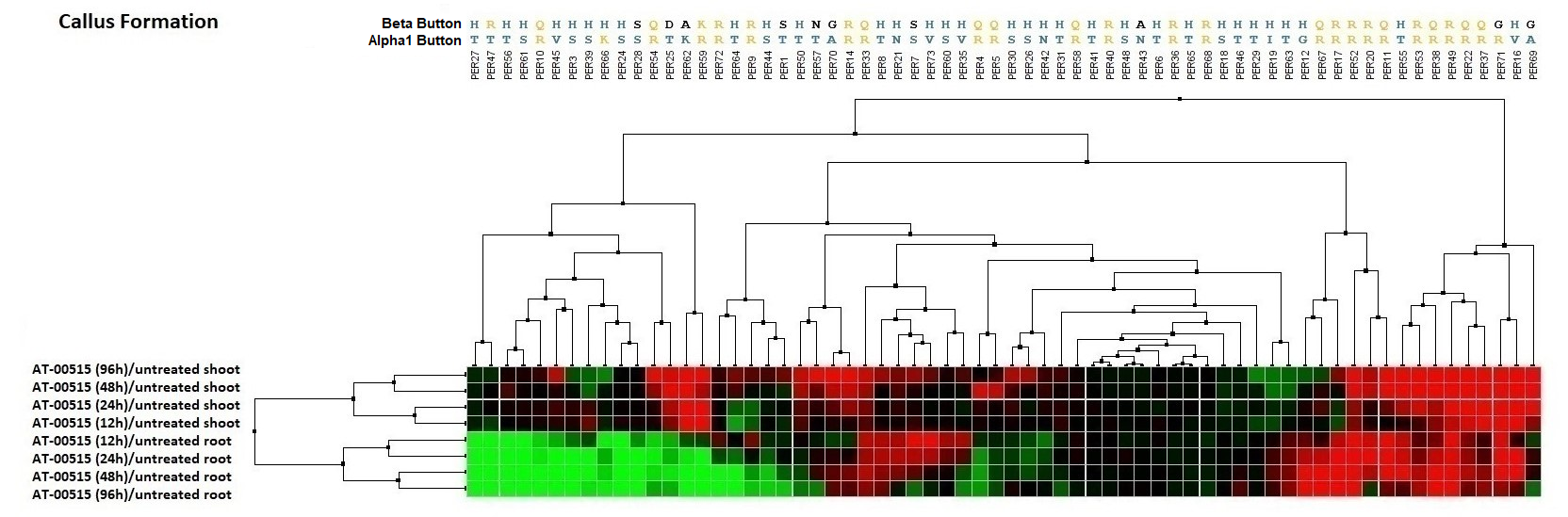

Supplement: Supplementary file 1 [file ijms-24-08297-s001.zip › Suppl_Figure_S1_Callus Formation 03 01 2023.png]

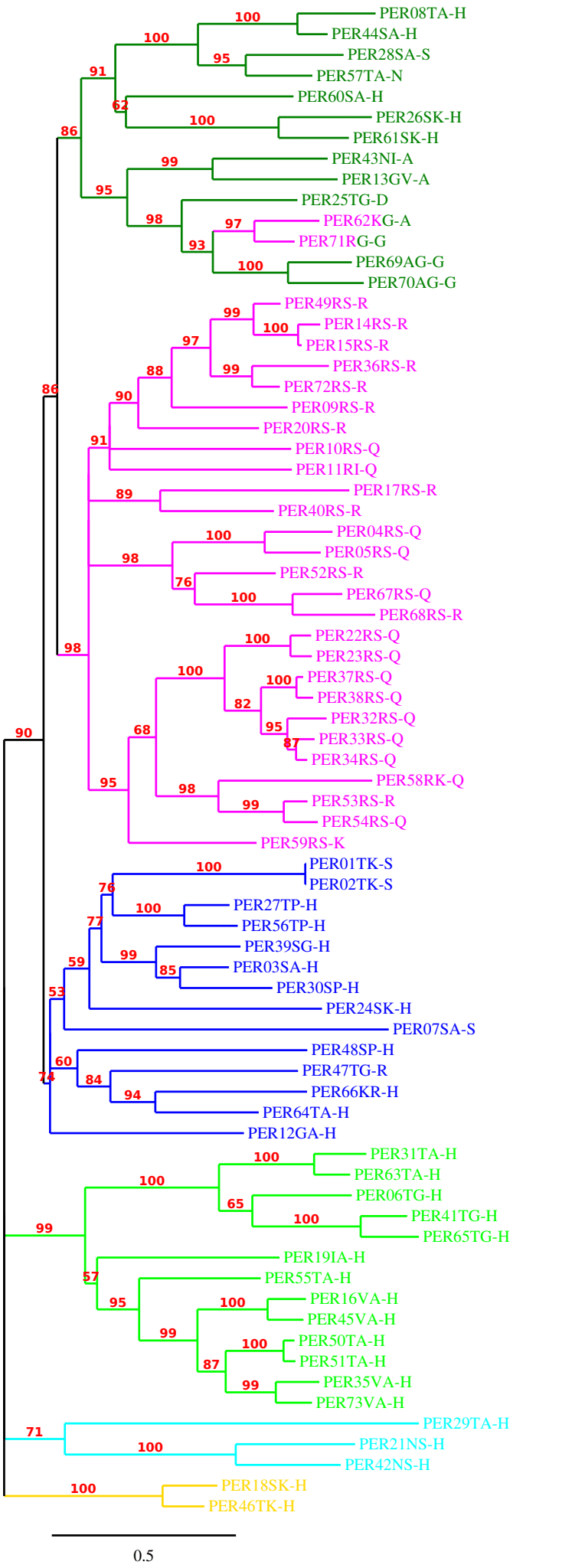

Supplement: Supplementary file 1 [file ijms-24-08297-s001.zip › Suppl_Figure_S2_phylo_tree Phylogram 03 01 2023 Version 0.3.png]

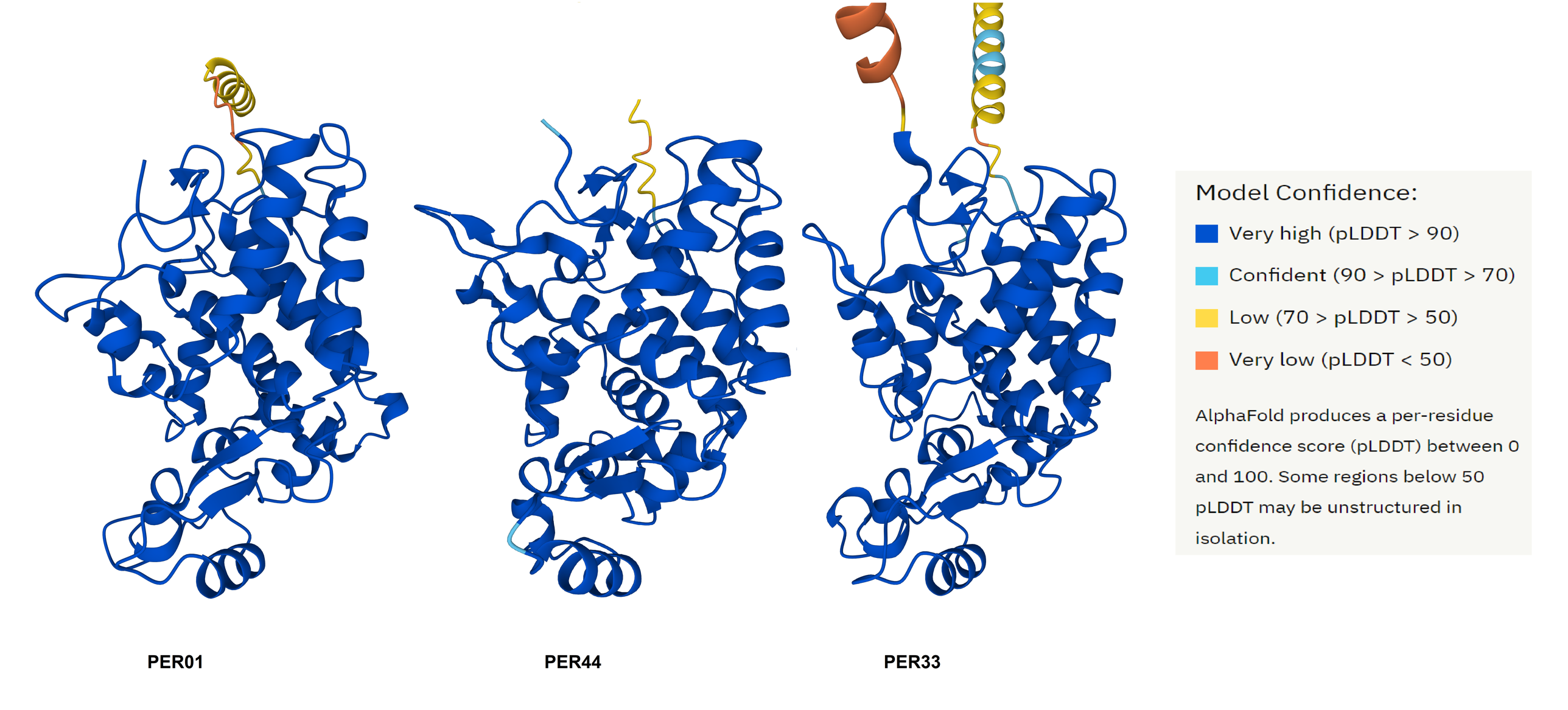

Supplement: Supplementary file 1 [file ijms-24-08297-s001.zip › Suppl_Figure_S3_AlphaFold Confidence 02 23 2023 Version 0.3.png]

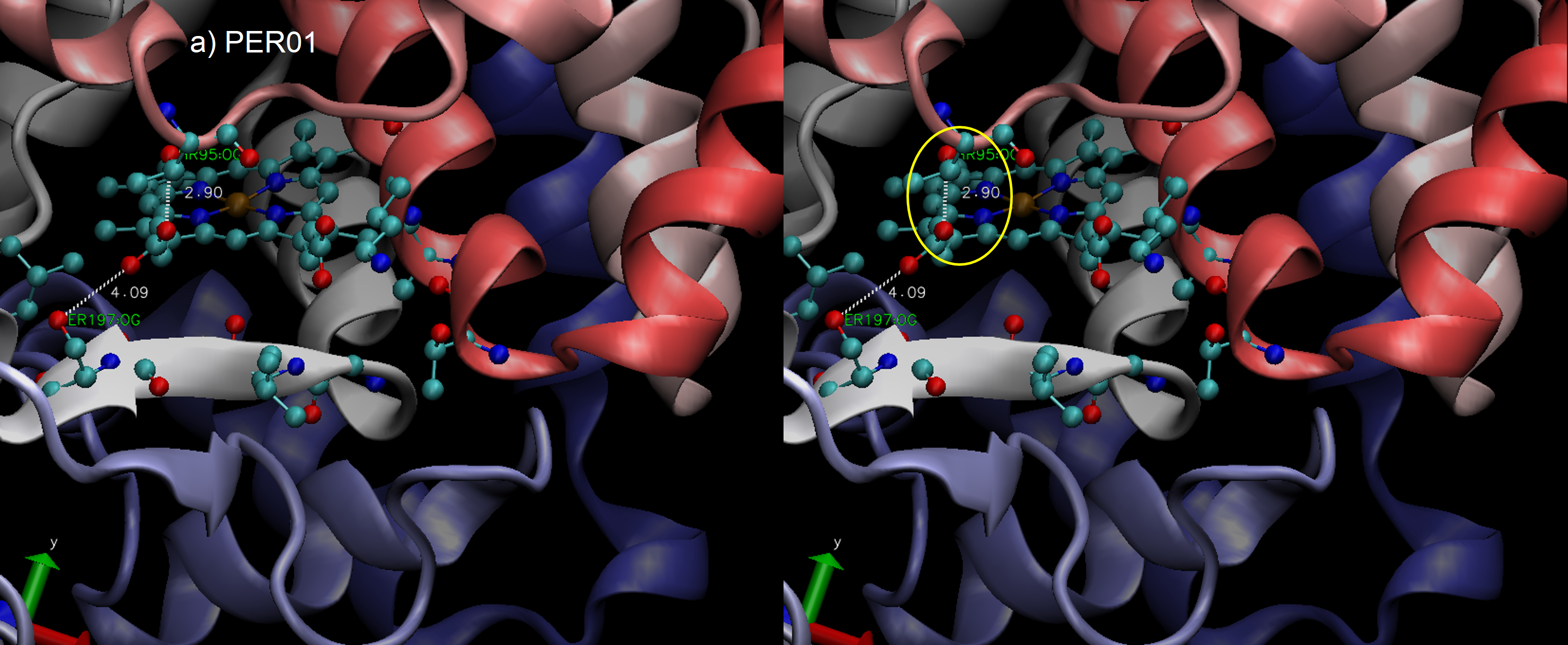

Supplement: Supplementary file 1 [file ijms-24-08297-s001.zip › Suppl_Figure_S4a-PER01.png]

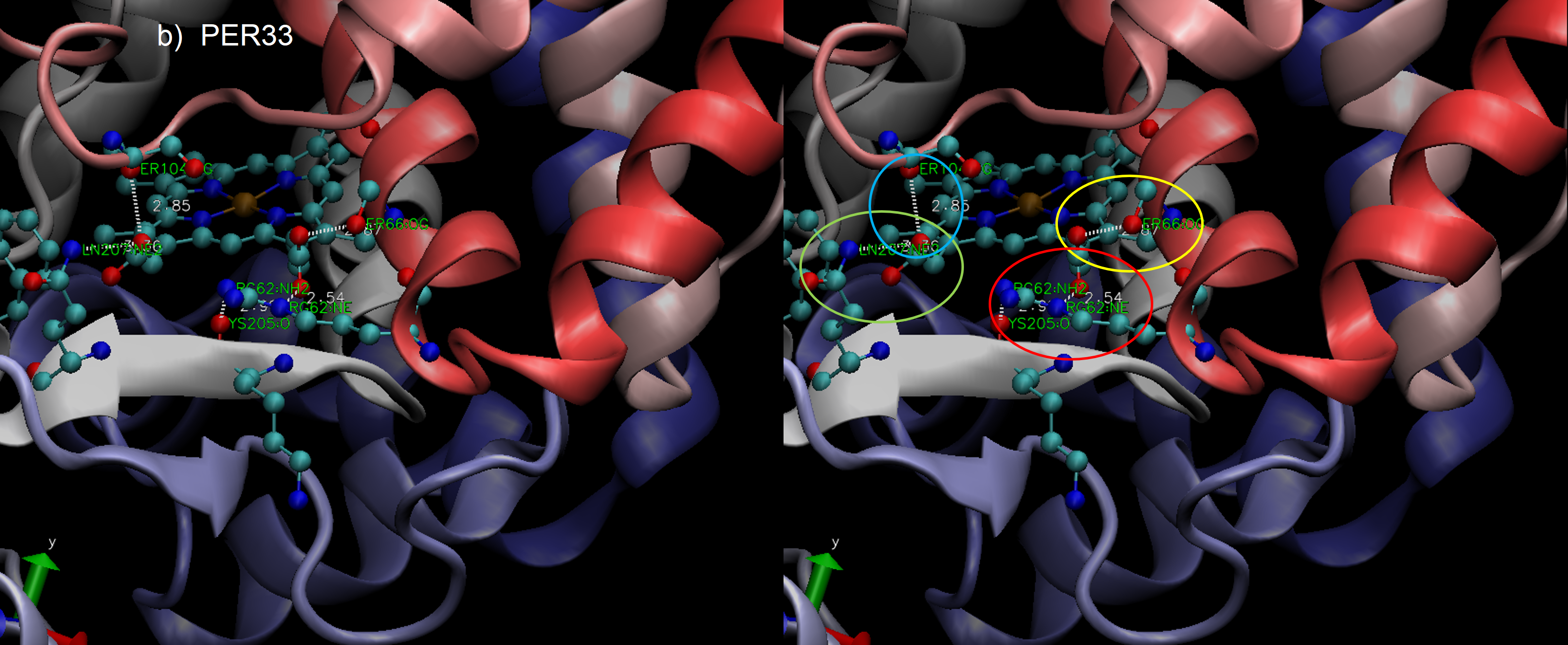

Supplement: Supplementary file 1 [file ijms-24-08297-s001.zip › Suppl_Figure_S4b-PER33.png]

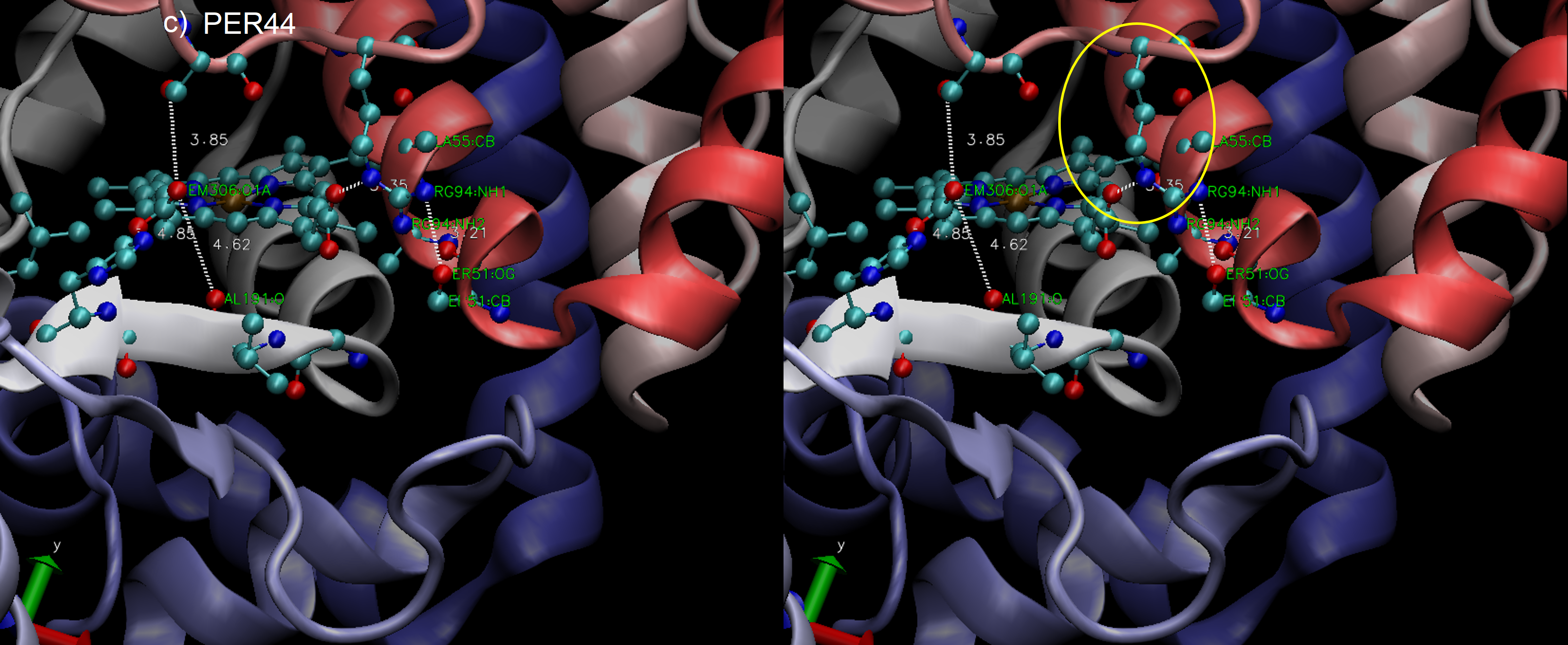

Supplement: Supplementary file 1 [file ijms-24-08297-s001.zip › Suppl_Figure_S4c-PER44.png]

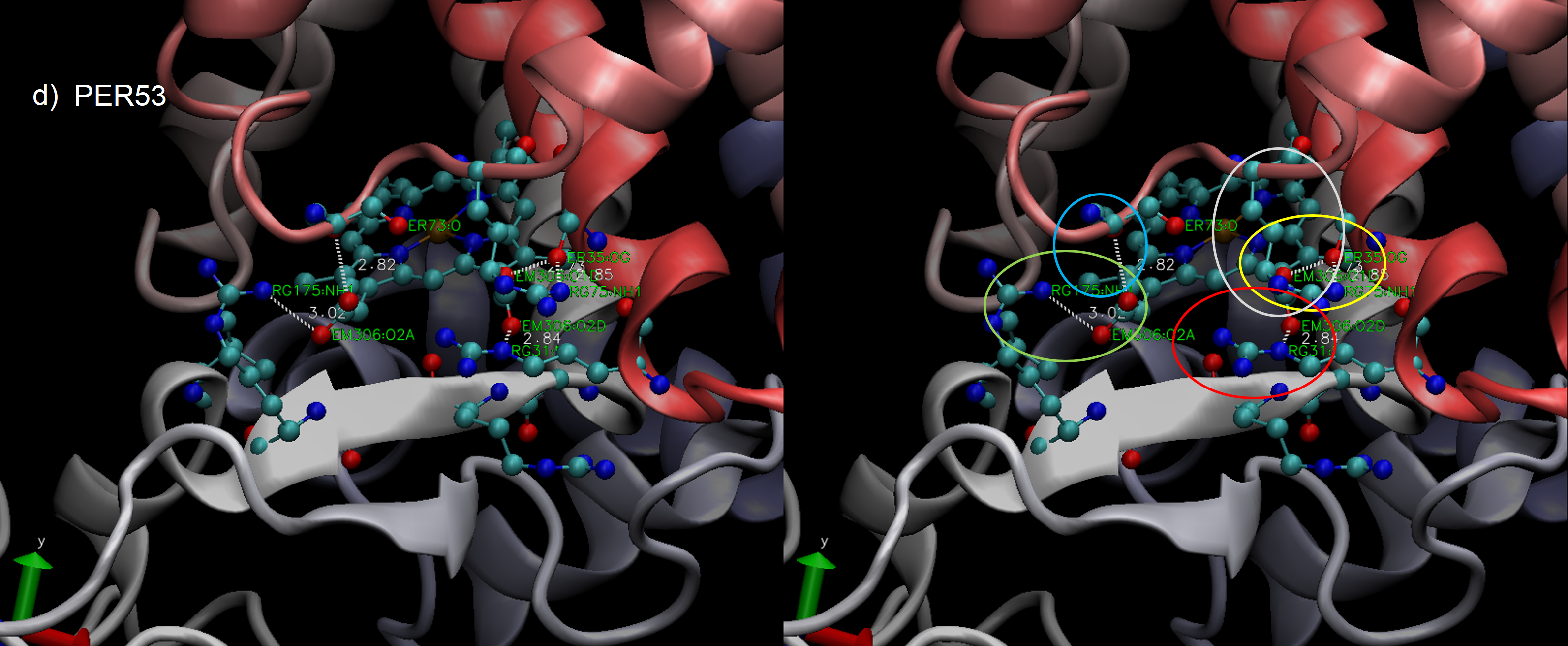

Supplement: Supplementary file 1 [file ijms-24-08297-s001.zip › Suppl_Figure_S4d-PER53.png]

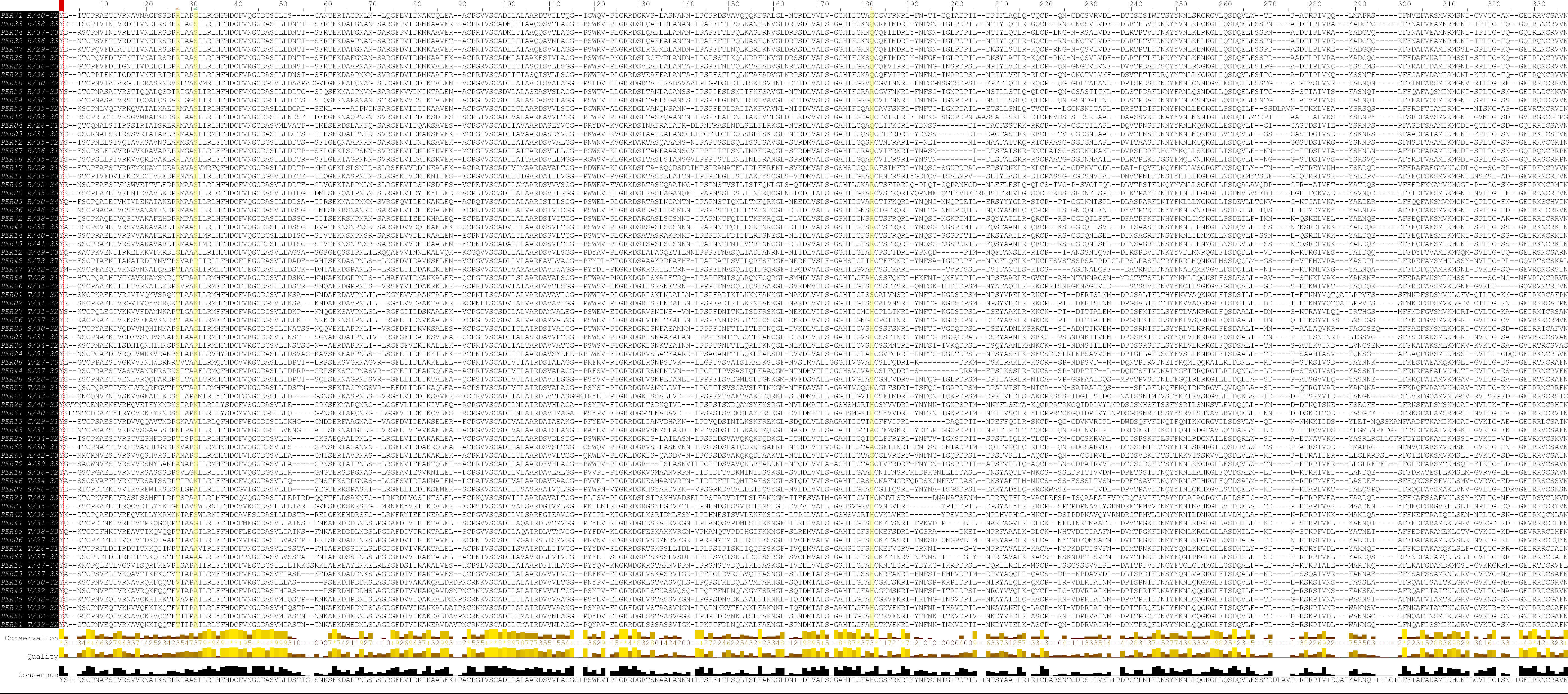

Supplement: Supplementary file 1 [file ijms-24-08297-s001.zip › Suppl_Figure_S5_Full Multiple Sequence Alignment-withButtons.png]
